# A Ranking of the Most Common Maternal COVID-19 Symptoms: A Systematic Review

**DOI:** 10.3389/fmed.2022.865134

**Published:** 2022-06-14

**Authors:** Melissa Chao, Carlo Menon, Mohamed Elgendi

**Affiliations:** ^1^Department of Medicine, Faculty of Medicine, University of British Columbia, Vancouver, BC, Canada; ^2^Biomedical and Mobile Health Technology Laboratory, Department of Health Sciences and Technology, Zurich, Switzerland

**Keywords:** signs and symptoms, COVID-19 management, screening tools, diagnosis, self-diagnosis, self-check assessment, early diagnosis

## Abstract

As the coronavirus disease 2019 (COVID-19) continues to devastate health systems worldwide, there is particular concern over the health and safety of one high-risk group, pregnant women, due to their altered immune systems. Since health workers regularly rely on symptoms to inform clinical treatment, it became critical to maintain a ranked list of COVID-19 symptoms specific to pregnant women. This systematic review investigated the prevalence of common COVID-19 symptoms in pregnant women and compared the ranked list of symptoms to articles of various sizes. Articles were included if they discussed pregnant women diagnosed with COVID-19 using polymerase chain reaction testing, and women present symptoms of COVID-19 and were published between December 1, 2019, and December 1, 2021; while articles were excluded if they did not report on pregnant women with COVID-19 displaying symptoms of COVID-19. Articles were identified on OVID MedLine and Embase in January of 2022. The risk of bias and quality appraisal was assessed using a nine-item modified Scottish Intercollegiate Guidelines Network checklist for case-control studies. The search results included 78 articles that described 41,513 pregnant women with 42 unique COVID-19 symptoms. When ranked, the most common symptoms were found to be cough (10,843 cases, 16.02%), fever (7,653 cases, 11.31%), myalgia (6,505 cases, 9.61%), headache (5,264 cases, 7.78%), and dyspnea (5,184 cases, 7.66%). When compared to other articles in the literature with sample sizes of *n* = 23,434, *n* = 8,207, and *n* = 651, the ranking largely aligned with those in other articles with large sample sizes and did not align with the results of articles with small sample sizes. The symptom ranking may be used to inform testing for COVID-19 in the clinic. Research is rapidly evolving with the ongoing nature of the pandemic, challenging the generalizability of the results.

## Introduction

In December 2019, China reported atypical pneumonia cases in Wuhan, Hubei Province, to the World Health Organization (WHO) (1). The viral pathogen responsible was named severe acute respiratory syndrome coronavirus 2 (SARS-CoV-2) due to its resemblance to an earlier coronavirus (2). The fatal disease was termed coronavirus disease 2019 (COVID-19) and was later characterized as a pandemic due to its rapid worldwide spread (2). As health systems around the world struggled to contain the spread, many systems focused on protecting high-risk groups.

One such high-risk group is pregnant women due to their altered immune systems that make them particularly susceptible to SARS-CoV-2 infection, while clinical identification may be difficult because pregnant women may present different COVID-19 symptoms to their non-pregnant counterparts (3). Since clinicians have been routinely assessing patients for COVID-19 symptoms to inform biochemical testing, and symptoms may vary significantly in pregnant women, it was necessary to develop a list of common COVID-19 symptoms in pregnant women (3).

An early systematic review published in 2020 examined the effects of COVID-19 on maternal, perinatal, and neonatal outcomes of 324 pregnant women. The list of most common symptoms includes fever, cough, dyspnea/shortness of breath, fatigue, and myalgia (4). Another review article on 108 pregnancies with confirmed SARS-CoV-2 indicated that fever was the most common symptom upon admission, while cough was second (5).

Another review of 10,996 patients from 15 countries worldwide, reported that cough and fever were the most prevalent symptoms, with cough present in around half of the eligible cases (6). A more recent systematic review of 11,758 pregnant women found that every fatal case of COVID-19 presented with fever with or without cough. They reported that dyspnea and myalgia were the most common symptoms, presenting in about half of patients, while the sore throat and gastrointestinal symptoms were rare symptoms (7).

Another systematic review compared the symptoms of various severe coronaviruses, finding similar symptoms between coronaviruses. For SARS-CoV-2, symptoms in 17 patients observed that fever, cough, myalgia, and chills were the most common symptoms (8). In 2021, a review article of 30 systematic reviews explored the top 10 most common symptoms. This review of reviews indicated on mothers with COVID-19 indicates the most common symptom is cough, and fever is a close second (9).

This systematic review analyzed the most common COVID-19 symptoms in pregnant women. As well, this systematic review examined the literature that focuses on the COVID-19 symptoms experienced by pregnant women. Based on the literature search, implications for clinical practice were determined and discussed.

## Methods

A systematic review of COVID-19-derived symptoms in pregnant female populations was undertaken in adherence to the guidelines of the Preferred Reporting Items for Systematic reviews and Meta-Analyses (PRISMA) and the protocol was not registered (10). A systematic review of the literature consisted of four stages: (1) a database and manual search were performed to identify potential articles, (2) articles were reviewed according to the inclusion and exclusion criteria, (3) data was extracted from included eligible articles, and (4) the extracted data was analyzed.

### Database Search

Search terms were developed to identify examine that included COVID-19 disease, pregnancy, and maternal COVID-19 symptoms. Search terms to identify articles relating to pregnancy included pregnancy, pregnant women, and pregnant women. The search that reflected COVID-19 included search terms: COVID-19, SARS-CoV-2, and coronavirus pregnancy. Finally, search terms to identify symptoms were most common symptoms, most frequent symptoms, most frequently reported symptoms, most common signs, most frequent signs, and most frequently reported signs. Taken together, the search terms were: ((Pregnancy) OR (pregnant woman) OR (pregnant women)) AND ((COVID-19) OR (SARS-CoV-2) OR (coronavirus pregnancy)) AND ((most common symptoms) OR (most frequent symptoms) OR (most frequently reported symptoms) OR (most common signs) OR (most frequent signs) OR (most frequently reported signs)).

Subsequently, the search terms were deployed in PubMed and Embase databases, collecting articles published between December 1, 2019, and December 1, 2021. Finally, a manual search was performed using the references section of relevant included articles.

### Inclusion and Exclusion Criteria

The titles and abstracts were screened to identify eligible articles. The inclusion criteria were as follows: (1) full-text available completely in English; (2) original articles, case reports, select letters to the editor, select commentaries, select communications, or pre-print articles; (3) published between December 1, 2019, and December 1, 2021; (4) included pregnant women diagnosed with COVID-19 using RT-PCR; (5) included pregnant women in any gestational trimester; (6) included pregnant women of any maternal age; and (7) had a relevant topic to COVID-19 symptoms and pregnant women.

The exclusion criteria were as follows: (1) not written in English; (2) subjects were not pregnant women; (3) subjects who were diagnosed with COVID-19 using methods other than RT-PCR; (4) did not report maternal COVID-19 symptoms; (5) incompatible article type, including review articles, guidelines, select communications, select commentaries, and select letters to the editors; and (6) covered an unrelated topic.

### Article Review

All identified articles were populated on Microsoft Excel and duplicates were manually removed. Subsequently, the article review phase consisted of three steps: (1) title and abstract screening according to the inclusion and exclusion criteria to assess eligibility, (2) full-text review according to the inclusion and exclusion criteria, (3) data extraction of relevant outcomes of interest according to from included articles. The article review was performed by MC and ME using Microsoft Excel (11).

### Data Extraction

During the data extraction phase, the reviewers used Microsoft Excel to capture outcomes of interest as described in the original work and the Supplementary Material section (11). Outcomes of interest included maternal COVID-19 status as indicated by a positive RT-PCR test result of any associated maternal COVID-19 symptoms. No data was extracted from individual mothers who were asymptomatic or did not have symptoms for any of the articles.

Multiple terms were classified under one unified term if the terms reflected the same clinical presentation; for example, “runny nose” and “rhinorrhea” were classified as “rhinorrhea,” and “lack of taste” and “ageusia” were both classified as “ageusia.” Additionally, multiple symptoms that were classified together in the original work were separated by the reviewers; for example, when an original work reported “lack of taste or smell,” the reviewers reported this as both a “lack of taste” and a “lack of smell,” and “nausea or vomiting” was reported as both “nausea” and “vomiting.” Once data was extracted for each of the included original works, the reviewers calculated the sum of each unique symptom.

### Data Analysis

Using Microsoft Excel, reported data on each symptom was aggregated, allowing the total number of symptoms and the confidence intervals to be calculated. Subsequently, the total number of symptoms was aggregated and ranked in order from most frequent to least frequent. For each symptom, the 95% confidence ranges were calculated using the number of women from each included article.

The resulting symptom ranking was presented compared with other relevant articles reporting a ranking of maternal COVID-19 symptoms; a comparison of the rankings was made with articles with sample sizes *n* = 23,434, *n* = 8,207, and *n* = 651 (12–14).

### Risk of Bias Assessment

Two raters rated the included articles, using a modified Scottish Intercollegiate Guidelines Network (SIGN) Checklist for Case-Control studies (15). Based on the question under investigation, the checklist was modified to include nine items for evaluation: Sections 1.1, 1.8, 1.9, 1.10, 1.11, 2.1, 2.2, 2.3, and 2.4.

The rating was performed in three steps. First, raters would evaluate citations and any disagreements were resolved by discussion. Citations were assessed on a three-point scale of “yes,” “no,” and “can't say” for each of the nine items. Then, for Section Database search, articles that were rated as high quality “++,” articles met the majority of criteria met, with little or no risk of bias, while articles rated as acceptable “+” had most criteria met with some flaws in the study design with an associated risk of bias. Articles rated as “0” were of low quality and failed to meet most criteria or had significant flaws relating to key aspects of the study design. Finally, studies were filtered according to their ability to minimize risk. Articles that were labeled as high quality or acceptable quality were retained, while those of unacceptable quality were rejected.

## Results

### Article Review and Selection Process

**Figure 1:**
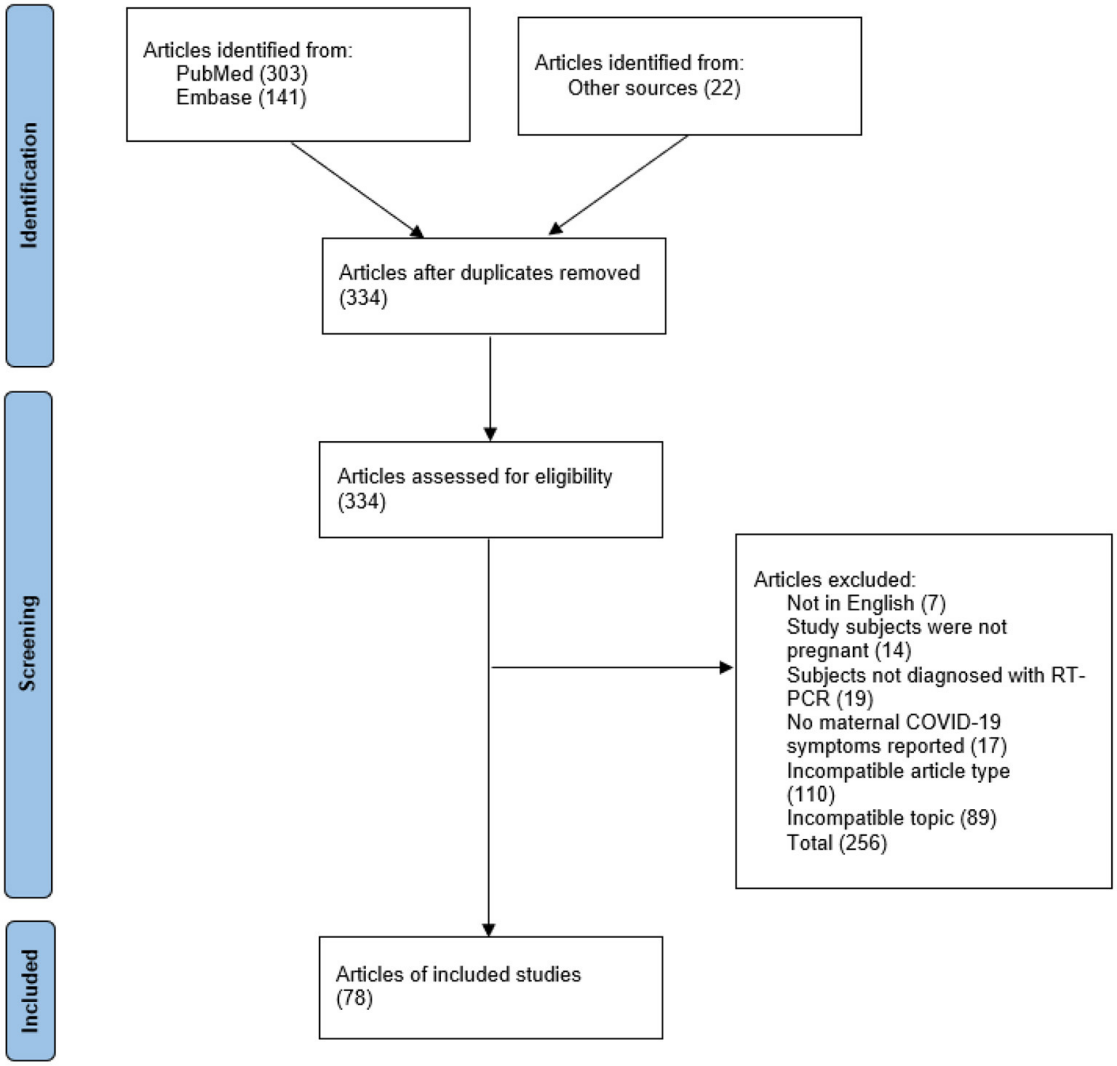
The process used to identify, screen, and verify the eligibility of articles.

The flow chart presented in Figure 1 shows the stages of the article review and selection process. A total of 303 articles were retrieved from PubMed, and 141 were retrieved from Embase, resulting in a pool of 444 articles. Once duplicates were removed, 312 unique articles remained. Then, the 312 articles were filtered according to the exclusion criteria, and 256 articles were excluded. This left 56 articles that met the search criteria. Additionally, 22 articles were manually retrieved from PubMed and Embase. In total, 78 articles met the inclusion criteria.

### Ranking of the Top 20 COVID-19 Symptoms

There were 42 unique COVID-19 symptoms identified from 67,665 total COVID-19 symptoms, which were observed in 41,513 RT-PCR-diagnosed pregnant women. The top five aggregated symptoms were found to be cough (10,843 cases, 16.02%), fever (7,653 cases, 11.31%), myalgia (6,505 cases, 9.61%), headache (5,264 cases, 7.78%), and dyspnea (5,184 cases, 7.66%) (Table 1).

**Table 1:** The top 20 most common COVID-19 symptoms in pregnant women.

**Rank**	**Symptom**	**Number of cases (%)**	**95% Confidence range**
1	Cough	10,843 (16.02%)	176.90–151.68
2	Fever	7,653 (11.31%)	117.10–98.47
3	Myalgia	6,505 (9.61%)	169.64–140.12
4	Headache	5,264 (7.78%)	233.95–187.17
5	Dyspnea	5,184 (7.66%)	99.50–79.26
6	Chills	3,853 (5.69%)	343.80–298.37
7	Sore throat	3,837 (5.67%)	140.18–107.37
8	Anosmia	3,513 (5.19%)	116.02–90.63
9	Ageusia	3,483 (5.15%)	138.36–110.43
10	Nausea	3,120 (4.61%)	226.25–189.75
11	Vomit	3,112 (4.60%)	200.39–165.73
12	Diarrhea	2,398 (3.54%)	72.96–53.25
13	Rhinorrhea	2,208 (3.26%)	128.60–103.82
14	Fatigue	2,181 (3.22%)	83.18–62.22
15	Abdominal pain	1,269 (1.88%)	110.88–84.35
16	Chest pain	849 (1.25%)	64.32–48.88
17	Nasal congestion	404 (0.60%)	39.02–28.32
18	Expectoration	237 (0.35%)	48.30–30.70
19	Respiratory discomfort	220 (0.33%)	N/A^*^
20	Respiratory distress	201 (0.30%)	46.58–33.82

* *N/A indicates no confidence range could be determined from the data*.

### Comparison of Ranked COVID-19 Symptoms in Pregnant Women

When the symptom ranking was compared to that in articles with different sample sizes, the results varied. In the three articles with large sample sizes, fever was ranked as the second most common symptom, while cough was ranked first in three of the four articles.

The articles included in this comparison were specifically selected to provide variation in sample size to emphasize the impact that sample size has on symptom ranking. The sample sizes were *n* = 651, *n* = 8,207, and *n* = 23,434 (12–14). As the sample size of the other articles approached that of this systematic review, the order of the COVID-19 symptoms approached the order found in this systematic review (Table 2).

**Table 2:** Top 10 most frequent symptoms across articles of different sample sizes.

**Symptom**	**Article 4** **(*n* = 651)**	**Article 3** **(*n* = 8,207)**	**Article 2** **(*n* = 23,434)**	**This review** **(*n* = 42,710)**
Cough	2	1	1	1
Fever	1	2	2	2
Myalgia	9	3	3	3
Headache	N/A	5	5	4
Dyspnea	3	6	6	5
Sore throat	8	7	7	6
Chills	N/A	4	4	7
Anosmia	7*	12*	12*	8
Ageusia	7*	12*	12*	9
Nausea	12**	9**	9**	10
Vomiting	12**	9**	9**	11
Diarrhea	11	8	8	12
Rhinorrhea	N/A	11	11	13
Fatigue	10	N/A	13	14
Abdominal pain	13	10	10	15
Chest pain	6	N/A	15	16
Nasal congestion	N/A	N/A	N/A	17
Expectoration	N/A	N/A	N/A	18
Respiratory discomfort	N/A	N/A	N/A	19
Respiratory distress	N/A	N/A	N/A	20

^*^Reported as anosmia or ageusia. ^**^Reported as nausea or vomiting.

## Discussion

### Review of the Literature

According to a literature search, this is the only systematic review of this size that ranks the frequency of COVID-19 symptoms in pregnant women diagnosed with COVID-19. Furthermore, it encompasses a larger sample size than any other original or type of review article on the frequency of COVID-19 symptoms in pregnant women.

This systematic review builds on the work of a clinical article by updating a list of 42 unique COVID-19 symptoms derived from a larger sample size of 41,513 pregnant women with 67,665 COVID-19 symptoms reported over 24 months (16). The list of COVID-19 symptoms reported in that clinical article by Ashraf et al. deviated from the ranking of COVID-19 symptoms presented in this systematic review. Here, the cough was the top symptom; however, in the clinical article by Ashraf et al., fever, shortness of breath, and fatigue were more common (16).

Amongst articles of various sample sizes, there is some disagreement, where many articles reported fever as the most common symptom. Some reported cough as the most common symptom, which agrees with the findings of this systematic review (17–23). As the sample size increases, the ranking of COVID-19 symptoms approaches the ranking presented in this paper. However, it should be noted that articles with large sample sizes contributed more to the ranking presented in this systematic review.

Indeed, as more articles report the symptoms of pregnant mothers, the sample size increase and it is anticipated that the ranking will change. This is especially true in the case of new variants, which may increase the prevalence of certain symptoms over others and alter the ranking. As it stands, the list of different symptoms is compressive; however future SARS-CoV-2 variants may cause symptoms not included in this list or alter the ranking of symptoms.

### Clinical Assessment Based on Presentation of COVID-19 Symptoms

It has been well-documented in the literature that pregnant women are less likely to manifest typical symptoms of COVID-19 and more likely to be admitted to the intensive care unit compared to non-pregnant women; this indicates that there is a critical need for clinicians to effectively screen pregnant women for COVID-19 (24). Below, clinical considerations for the top five COVID-19 symptoms in pregnant women are described.

#### 1. Cough

The literature agrees that cough is less common in pregnant women than in non-pregnant women (22–24). However, it is one of the most easily identified symptoms in COVID-19 patients since it is not only one of the most common COVID-19 symptoms but also presents early in the pathological timeline (23–26).

As cough may be qualified with various clinical descriptors (e.g., dry cough or non-productive cough), it is important to include all forms of cough, to allow clinicians to quickly screen for it. Therefore, this systematic review clusters various forms of cough as simply “cough” (27).

#### 2. Fever

Although fever is one of the most common symptoms and is considered an early symptom of COVID-19 in pregnant women, there is agreement that fever is less common in pregnant women than in non-pregnant women (22, 23, 26, 28, 29). To complicate matters, unlike in non-pregnant women, fever can be identified both ante- and postpartum in mothers, but both forms are classified broadly as “fever” in this systematic review.

In the clinic, fever is typically identified when a patient presents with a temperature between 37.6 and 39.0 °C via the use of a temperature gun or thermometer (28). Although fever is a common COVID-19 symptom, it may be caused by a variety of factors other than viral infection. Therefore, clinicians should evaluate for fever and symptoms of respiratory infection during screening (4, 5, 30).

#### 3. Myalgia

This systematic review categorized myalgia, muscle pain, and muscle soreness as “myalgia” due to their clinical similarities. “Chest pain,” “abdominal pain,” “back pain,” and “joint pain” were classified separately due to reporting practices and clinical distinction. Analogous to the situation with other symptoms, pregnant women are less likely to manifest symptoms of myalgia than non-pregnant women (24).

In agreement with the literature, the findings of this systematic review show that myalgia is a common COVID-19 symptom frequently used as an indicator for screening, but that it is not one of the most common symptoms (31–33). Along with other symptoms, myalgia is associated with adverse pregnancy outcomes (e.g., high preterm birth rates and adverse pregnancy events), and therefore it is a significant clinical symptom that can be used as a risk assessment tool in pregnant women (33).

#### 4. Headache

As with many other infections, headache is a common symptom identified in both pregnant and non-pregnant patients with confirmed SARS-CoV-2 infection (34, 35). In COVID-19 patients, headaches have been localized to specific lobes within the brain or found to be diffused among many regions of the brain (36).

Another systematic review reported that headache was present with the disease but did not strongly associate with more severe disease (37). Compared to non-pregnant women, a higher proportion of pregnant women were found to experience headaches during the disease. Moreover, headache during pregnancy or postpartum was found to be worse in mothers with a chronic headache or an increase in severity (e.g., pain or pattern) compared to usual (38).

#### 5. Dyspnea

Dyspnea (or shortness of breath) is a common symptom, although less common than fever and cough (4, 30, 39). It has been reported that the severity of dyspnea upon presentation at hospitals is correlated with more severe pathology and subsequent maternal death (40). In the clinic, dyspnea may be challenging to discern from gestational dyspnea owning to higher maternal demands for oxygen for metabolism, gestational anemia, and fetal respiration (30). Although shortness of breath does not appear in all mothers, the frequency at which it appears suggests that an initial screening should include shortness of breath (16, 30).

**Figure 2:**
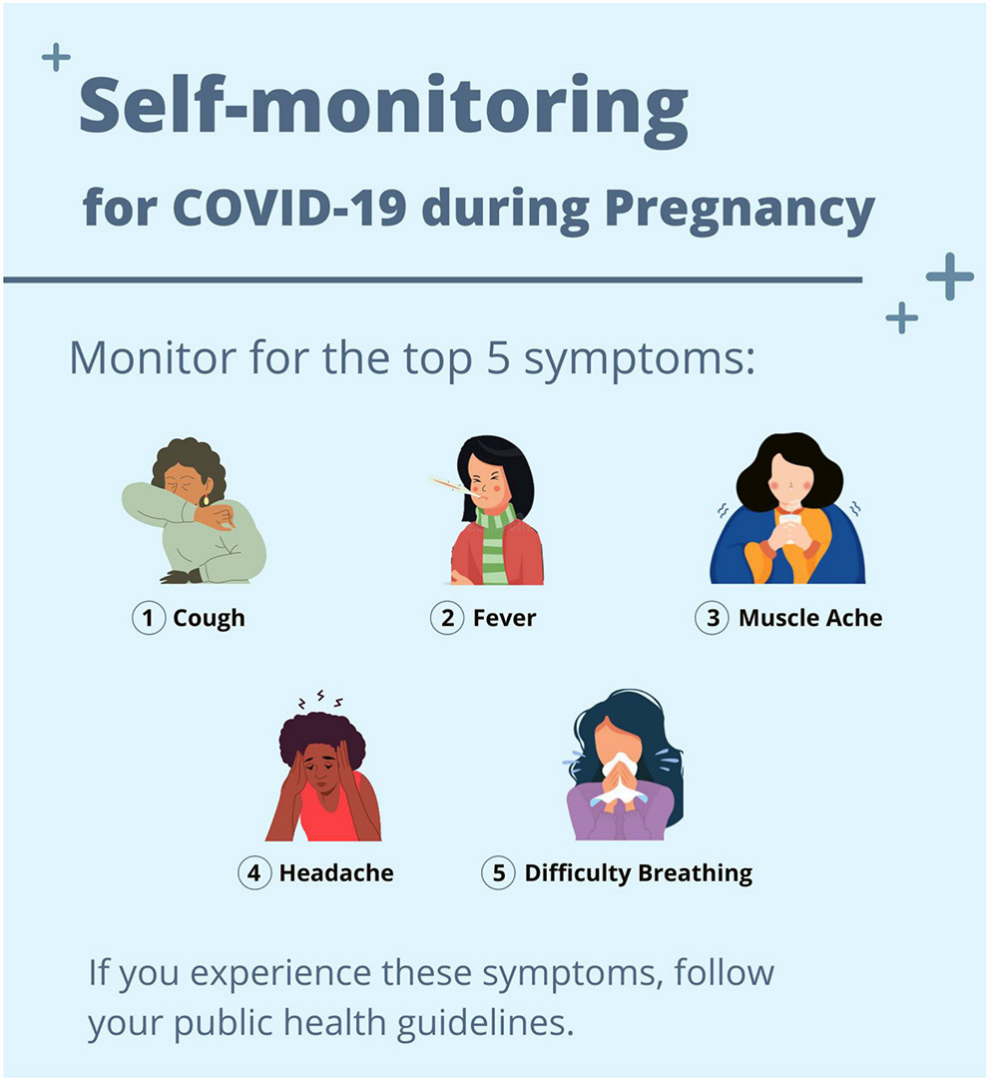
An infographic educates pregnant women to monitor for the top 5 symptoms of COVID-19.

Additionally, an infographic that educates pregnant women to monitor for the top five symptoms of COVID-19 is presented below (Figure 2). This figure was adapted from the WHO's COVID-19: symptoms and severity infographics, specifically tailoring recommendations to pregnant women (41).

### Limitations

One limitation of this systematic review is its ability to discern the etiology of certain symptoms as COVID-19 symptoms rather than derived from pregnancy. For example, both fatigue and muscle pain are typically observed in pregnant women and non-pregnant patients with COVID-19. Because these symptoms have been included in the systematic review, the sample numbers for these symptoms may be inflated. Therefore, clinicians must be wary of multiple possible etiologies for symptoms and must screen for multiple symptoms of COVID-19.

Additionally, the symptoms ranking included all SARS-CoV-2 variants across multiple time points from countries all around the world. Since the symptomology of COVID-19 varies according to the variant and within different populations, the result of this systematic review cannot be generalized. This data is crucial for understanding the clinical presentation of COVID-19 symptoms in pregnant women. Finally, the ongoing nature of the pandemic poses challenges to the generalizability of the results as research is rapidly evolving.

### Future Work

As this systematic review consolidates data from all variants, future studies should aggregate symptom data according to variants, which would enable the comparison of clinical appearances of variants to help inform more effective treatments. Further resolution may be provided if symptomology was compared between various countries at one-time point to develop a profile of the symptoms in pregnant women.

In addition, future studies may build on this work by comparing COVID-19 symptoms in women at different stages of pregnancy and post-partum women, to provide more resolution to the pathophysiological course.

## Conclusion

In summary, 42 unique COVID-19 symptoms were identified in 41,513 RT-PCR-diagnosed pregnant women. The top five COVID-19 symptoms in pregnant women were found to be cough, fever, muscle pain, headache, and shortness of breath. A list of ranked COVID-19 symptoms in pregnant women can be used as part of the clinical assessment when determining whether biochemical screening is required.

## Data Availability Statement

The original contributions presented in the study are included in the article/Supplementary Material, further inquiries can be directed to the corresponding author.

## Author Contributions

MC, CM, and ME conceived the study. All authors approved the final manuscript.

## Conflict of Interest

The authors declare that the research was conducted in the absence of any commercial or financial relationships that could be construed as a potential conflict of interest.

## Publisher's Note

All claims expressed in this article are solely those of the authors and do not necessarily represent those of their affiliated organizations, or those of the publisher, the editors and the reviewers. Any product that may be evaluated in this article, or claim that may be made by its manufacturer, is not guaranteed or endorsed by the publisher.
