# Supplementary material for: A Ranking of the Most Common Maternal COVID-19 Symptoms: A Systematic Review
Source: Front Med (Lausanne). 2022 Jun 14;9:865134. doi: 10.3389/fmed.2022.865134 (PMC9239403; doi:10.3389/fmed.2022.865134)
Supplement: Supplementary file 1 [file Data_Sheet_1.pdf]

Supplementary Information

Supplementary Information 1: All 42 maternal COVID-19 symptoms.

Symptom	n (%)	95% CL Range
Fever	7653 (11.31%)	117.10—98.47
Cough	7773 (11.42%)	176.90—151.68
Dyspnea	6556 (9.63%)	99.50—79.26
Fatigue	5283 (7.76%)	83.18—62.22
Anosmia	5236 (7.69%)	116.02—90.63
Myalgia	3866 (5.68%)	169.64—140.12
Sore throat	3857 (5.67%)	140.18—107.37
Headache	3507 (5.15%)	233.95—187.17
Diarrhea	3477 (5.11%)	72.96—53.25
Chest pain	3131 (4.60%)	64.32—48.88
Rhinorrhea	3112 (4.57%)	128.60—103.82
Malaise	2406 (3.54%)	14.67—9.76
Pneumonia	2213 (3.25%)	20.28—11.72
Chills	2181 (3.20%)	343.80—298.37
Nausea	1272 (1.87%)	226.25—189.75
Nasal congestion	861 (1.27%)	39.02—28.32
Vomit	429 (0.63%)	200.39—165.73
Ageusia	237 (0.35%)	138.36—110.43
Hypoxia	220 (0.32%)	35.75—23.85
Abdominal pain	201 (0.30%)	110.88—84.35
Tachycardia	187 (0.27%)	90.07—67.93
Conjunctivitis	172 (0.25%)	5.51—2.49
Respiratory distress	158 (0.23%)	46.58—33.82
Rhinitis	157 (0.23%)	5.35—1.65
General Gi symptoms	149 (0.22%)	11.40—6.31
Rash	96 (0.14%)	24.72—12.88
Back pain	94 (0.14%)	N/A
Hypothermia	65 (0.10%)	N/A
General neurological symptoms	60 (0.09%)	106.76—80.24
Odynophagia	48 (0.07%)	20.60—10.40
Joint pain	31 (0.05%)	28.85—19.15
Confusion	28 (0.04%)	4.60—1.40
Loss of appetite	26 (0.04%)	13.17—5.49
Chest fullness	16 (0.02%)	N/A
Red eye	7 (0.01%)	14.92—11.08
Dizziness	6 (0.01%)	2.07—0.93

Expectoration	5 (0.01%)	48.30—30.70
Earache	3 (0%)	N/A
Respiratory discomfort	2 (0%)	N/A
Sneeze	2 (0%)	N/A
Sweating	2 (0%)	N/A
Wheezing	1 (0%)	N/A

Supplementary Information 2: Reference list of 78 articles included in systematic review.

1. Hosier, H. *et al.* SARS-CoV-2 infection of the placenta. *Journal of Clinical Investigation* **130**, 4947–4953 (2020).
2. Facchetti, F. *et al.* SARS-CoV2 vertical transmission with adverse effects on the newborn revealed through integrated immunohistochemical, electron microscopy and molecular analyses of Placenta. *EBioMedicine* **59**, 102951–102959 (2020).
3. Liu, F. *et al.* Clinico-radiological features and outcomes in pregnant women with COVID-19 pneumonia compared with age-matched non-pregnant women. *Infection and Drug Resistance* **13**, 2845–2854 (2020).
4. Mosayebi, Z. *et al.* Clinical findings, laboratory assessment, and outcomes of 44 infants born to mothers with confirmed or suspected COVID-19: A multicenter cohort study. *Iranian Journal of Pediatrics* **31**, (2021).
5. Li, N. *et al.* Maternal and Neonatal Outcomes of Pregnant Women with Coronavirus Disease 2019 (COVID-19) Pneumonia: A Case-Control Study. *Clinical Infectious Diseases* **71**, 2035–2041 (2020).
6. Hernández-Cruz, R. G. *et al.* Clinical characteristics and risk factors for SARS-CoV-2 infection in pregnant women attending a third level reference center in Mexico City. *Journal of Maternal-Fetal and Neonatal Medicine* 1–5 (2021) doi:10.1080/14767058.2021.1902500.
7. Lokken, E. M. *et al.* Clinical characteristics of 46 pregnant women with a severe acute respiratory syndrome coronavirus 2 infection in Washington State. *American Journal of Obstetrics and Gynecology* **223**, 911.e1-911.e14 (2020).
8. Arakaki, T. *et al.* Clinical characteristics of pregnant women with COVID-19 in Japan: a nationwide questionnaire survey. *BMC Pregnancy and Childbirth* **21**, (2021).
9. Ozsurmeli, M. *et al.* Clinical characteristics, maternal and neonatal outcomes of pregnant women with SARS-CoV-2 infection in Turkey. *Bratislava Medical Journal* **122**, 152–157 (2021).
10. Wei, L. *et al.* Clinical characteristics and outcomes of childbearing-age women with COVID-19 in Wuhan: Retrospective, single-center study. *Journal of Medical Internet Research* **22**, e19642 (2020).
11. Osaikhuwuomwan, J. *et al.* Clinical characteristics and outcomes for pregnant women diagnosed with covid-19 disease at the university of benin teaching hospital, benin city, nigeria. *Pan African Medical Journal* **39**, (2021).
12. Chen, H. *et al.* Clinical characteristics and intrauterine vertical transmission potential of COVID-19 infection in nine pregnant women: a retrospective review of medical records. *The Lancet* **395**, 809–815 (2020).
13. Zhu, H. *et al.* Clinical analysis of 10 neonates born to mothers with 2019-nCoV pneumonia. *Translational Pediatrics* **9**, 51–60 (2020).
14. Sena, G. R. *et al.* Clinical characteristics and mortality profile of COVID-19 patients aged less than 20 years old in Pernambuco – Brazil. *American Journal of Tropical Medicine and Hygiene* **104**, 1507–1512 (2021).
15. Chen, S. *et al.* Clinical analysis of pregnant women with 2019 novel coronavirus pneumonia. *Journal of Medical Virology* **92**, 1556–1561 (2020).
16. Gao, X. *et al.* Clinical and immunologic features among COVID-19–affected mother–infant pairs: antibodies to SARS-CoV-2 detected in breast milk. *New Microbes and New Infections* **37**, (2020).
17. Abedzadeh-Kalahroudi, M., Sehat, M., Vahedpour, Z., Talebian, P. & Haghighi, A. Clinical and obstetric characteristics of pregnant women with Covid-19: A case series study on 26 patients. *Taiwanese Journal of Obstetrics and Gynecology* **60**, 458–462 (2021).
18. Luo, Q., Yao, D., Xia, L., Cheng, Y. & Chen, H. Characteristics and Pregnancy Outcomes of Asymptomatic and Symptomatic Women with COVID-19: Lessons from Hospitals in Wuhan. *Journal of Infection in Developing Countries* **15**, 463–469 (2021).

19. Ellington, S. *et al.* Characteristics of Women of Reproductive Age with Laboratory-Confirmed SARS-CoV-2 Infection by Pregnancy Status-United States. *Morbidity and Mortality Weekly* **69**, 769–776 (2020).
20. Zumalave Grados, I. *et al.* Characteristics of SARS-CoV-2 infection in pregnant and puerperal women at Callao national hospital, Peru. *Revista Peruana de Ginecología y Obstetricia* **66**, (2020).
21. Zambrano, L. D. *et al.* Update: Characteristics of Symptomatic Women of Reproductive Age with Laboratory-Confirmed SARS-CoV-2 Infection by Pregnancy Status — United States, January 22–October 3, 2020. *Morbidity and Mortality Weekly Report* **69**, 1641–1648 (2020).
22. Vizheh, M. *et al.* Characteristics and outcomes of COVID-19 pneumonia in pregnancy compared with infected nonpregnant women. *Journal of Obstretics and Gynecology* **153**, 462–468 (2021).
23. Knight, M. *et al.* Characteristics and outcomes of pregnant women admitted to hospital with confirmed SARS-CoV-2 infection in UK: National population based cohort study. *British Medical Journal* **369**, (2020).
24. Dey, M. *et al.* Case report: Organizing Pneumonia in a Pregnant Woman with Rheumatoid Arthritis during COVID-19 Pandemic. *Annals of the Rheumatic Diseases* **80**, 1357.2-1358 (2021).
25. Guo, Y. *et al.* Case series of 20 pregnant women with 2019 novel coronavirus disease in Wuhan, China. *Journal of Obstetrics and Gynaecology Research* **47**, 1344–1352 (2021).
26. Oropeza Chávez, L. *et al.* A 34-Year-Old Woman with a Diamniotic Dichorionic Twin Pregnancy Presenting with an Erythematous and Papular Skin Rash Associated with SARS-CoV-2 Infection. *The American journal of case reports* **22**, e929489 (2021).
27. Pulinx, B. *et al.* Vertical transmission of SARS-CoV-2 infection and preterm birth. *European Journal of Clinical Microbiology and Infectious Diseases* **39**, 2441–2445 (2020).
28. Ibrahim, S. A. *et al.* ABO blood group, rhesus type and risk of COVID-19 in pregnant women. *American Journal of Obstetrics and Gynecology* **224**, S605–S605 (2021).
29. Moreno, S. C., To, J., Chun, H. & Ngai, I. M. Vertical Transmission of COVID-19 to the Neonate. *Infectious Diseases in Obstetrics and Gynecology* **2020**, (2020).
30. Rottenstreich, A. *et al.* Vaginal delivery in SARS-CoV-2-infected pregnant women in Israel: a multicenter prospective analysis. *Archives of Gynecology and Obstetrics* **303**, 1401–1405 (2021).
31. Sahin, D. *et al.* Updated experience of a tertiary pandemic center on 533 pregnant women with COVID-19 infection: A prospective cohort study from Turkey. *International Journal of Gynecology and Obstetrics* **152**, 328–334 (2021).
32. Soto-Torres, E., Hernandez-Andrade, E., Huntley, E., Mendez-Figueroa, H. & Blackwell, S. C. Ultrasound and Doppler findings in pregnant women with SARS-CoV-2 infection. *Ultrasound in Obstetrics and Gynecology* **58**, 111–120 (2021).
33. Tien-Chan, H. *et al.* Unexpected Cause of Postpartum Cardiac Arrest: Primary Mediastinal Large B-cell Lymphoma. *Chest* **160**, 1292A (2020).
34. Federici, L., Picone, O., Dreyfuss, Di. & Sibiude, J. Successful continuation of pregnancy in a patient with COVID-19-related ARDS. *British Medical Journal Case Reports* **13**, 3237511 (2020).
35. Barbero, P. *et al.* SARS-CoV-2 in pregnancy: characteristics and outcomes of hospitalized and non-hospitalized women due to COVID-19. *Journal of Maternal-Fetal and Neonatal Medicine* 1–7 (2020) doi:10.1080/14767058.2020.1793320.
36. London, V. *et al.* The Relationship between Status at Presentation and Outcomes among Pregnant Women with COVID-19. *American Journal of Perinatology* **37**, 991–994 (2020).
37. Hodžić, J., Muračević, B., Štimjanin, H., Iriškić, R. & Husika, M. Pregnancy outcomes of COVID-19 positive pregnant women at the Cantonal Hospital Zenica, Bosnia and Herzegovina. *Medicinski glasnik : official publication of the Medical Association of Zenica-Doboj Canton, Bosnia and Herzegovina* **19**, 334–337 (2022).
38. Roriguez Chavez, R. *et al.* Pregnant and Postpartum Women with Rheumatic Diseases and COVID-19: A Case Series. *Annals of the Rheumatic Diseases* **80**, 892–893 (2021).
39. Liu, D. *et al.* Pregnancy and perinatal outcomes of women with coronavirus disease (COVID-19) Pneumonia: A preliminary analysis. *American Journal of Roentgenology* **215**, 127–132 (2020).

40. Choudhary, A., Singh, V., Bharadwaj, M. & Barik, A. Pregnancy With SARS-CoV-2 Infection Complicated by Preeclampsia and Acute Fatty Liver of Pregnancy. *Cureus* **13**, (2021).
41. Mullins, E. *et al.* Pregnancy and neonatal outcomes of COVID-19: coreporting of common outcomes from PAN-COVID and AAP-SONPM registries. *Ultrasound in Obstetrics and Gynecology* **57**, 573–581 (2021).
42. Kim, H. K., Cho, Y. J. & Lee, S. Y. Neurological manifestations in patients with COVID-19: Experiences from the central infectious diseases hospital in South Korea. *Journal of Clinical Neurology (Korea)* **17**, 435–442 (2021).
43. Sanchez-Luna, M. *et al.* Neonates Born to Mothers With COVID-19: Data From the Spanish Society of Neonatology Registry. *Pediatrics* **147**, (2021).
44. Hcini, N. *et al.* Maternal, fetal and neonatal outcomes of large series of SARS-CoV-2 positive pregnancies in peripartum period: A single-center prospective comparative study. *European Journal of Obstetrics and Gynecology and Reproductive Biology* **257**, 11–18 (2021).
45. Shlomai, N. *et al.* Neonatal SARS-CoV-2 Infections in Breastfeeding Mothers. *American Academy of Pediatrics* **147**, (2021).
46. Kogutt, B. K. & Satin, A. J. Maternal mortality among women with coronavirus disease 2019 admitted to the intensive care unit. *American Journal of Obstetrics and Gynecology* **223**, 595-599.e1 (2020).
47. Saccone, G. *et al.* Maternal and perinatal outcomes of pregnant women with SARS-CoV-2 infection. *Ultrasound in Obstetrics and Gynecology* **57**, 232–241 (2021).
48. Mohr-Sasson, A. *et al.* Laboratory characteristics of pregnant compared to non-pregnant women infected with SARS-CoV-2. *Maternal-Fetal Medicine* **302**, 629–634 (2020).
49. Teixeira, M. de L. B. *et al.* Maternal and neonatal outcomes of sars-cov-2 infection in a cohort of pregnant women with comorbid disorders. *Viruses* **13**, (2021).
50. Ayed, A. *et al.* Maternal and perinatal characteristics and outcomes of pregnancies complicated with COVID-19 in Kuwait. *BMC Pregnancy and Childbirth* **20**, (2020).
51. Dawood, F. S. *et al.* Incidence, Clinical Characteristics, and Risk Factors of SARS-CoV-2 Infection among Pregnant Individuals in the United States. *Clinical infectious diseases: an official publication of the Infectious Diseases Society of America* (2021).
52. AlOmran, A., Almatawah, Y., al Sharit, B., Alsadah, Z. & Mousa, O. Infection Prevention and Control Challenges With First Pregnant Woman Diagnosed With COVID-19: A Case Report in Al Ahssa, Saudi Arabia. *Cureus* **12**, (2020).
53. Fabre, M. *et al.* Frequent Placental SARS-CoV-2 in Patients with COVID-19-Associated Hypertensive Disorders of Pregnancy. *Fetal Diagnosis and Therapy* **18**, 1–11 (2021).
54. Rosen, H. *et al.* Fetal and perinatal outcome following first and second trimester covid-19 infection: Evidence from a prospective cohort study. *Journal of Clinical Medicine* **10**, 2152 (2021).
55. Ronchi, A. *et al.* Evaluation of Rooming-in Practice for Neonates Born to Mothers with Severe Acute Respiratory Syndrome Coronavirus 2 Infection in Italy. *JAMA Pediatrics* **175**, 1–8 (2021).
56. Solís-García, G. *et al.* Epidemiology, management and risk of SARS-CoV-2 transmission in a cohort of newborns born to mothers diagnosed with COVID-19 infection. *Anales de Pediatría* **94**, 173–178 (2021).
57. Harel, L. *et al.* Does the presence of symptoms affect pregnancy outcomes in third trimester in women with SARS-CoV-2. *Journal of Maternal-Fetal and Neonatal Medicine* 1–8 (2021) doi:10.1080/14767058.2021.1956895.
58. Li, J. *et al.* Critically ill pregnant patient with COVID-19 and neonatal death within two hours of birth. *International Journal of Gynecology and Obstetrics* **150**, 126–128 (2020).
59. Metz, T. D. *et al.* Disease Severity and Perinatal Outcomes of Pregnant Patients With Coronavirus Disease 2019 (COVID-19). *Obstetrics and gynecology* **137**, 571–580 (2021).
60. Molina, E. O. *et al.* Covid-19 Infection in Symptomatic Pregnant Women at the Midpoint of the Pandemic in Spain: A Retrospective Analysis. *Ginekologia Polska* **91**, 755–763 (2020).
61. Sánchez, J. *et al.* COVID 19 and high pregnancy and perinatal complications in Panama. *Journal of Maternal-Fetal and Neonatal Medicine* **150**, 126–128 (2021).

62. Ames, J. L. *et al.* COVID-19 prevalence, symptoms, and sociodemographic disparities in infection among insured pregnant women in Northern California. *PLoS ONE* **16**, e0256891 (2021).
63. Damar Çakırca, T. *et al.* COVID-19 infection in pregnancy: a single center experience with 75 cases. *Ginekologia Polska* (2021) doi:10.5603/gp.a2021.0118.
64. Alasia D, Vo, O., Jn, I. & Stanley P. COVID-19 and Pregnancy: A Prospective Descriptive Study from a Tertiary Hospital in Nigeria. *West African Journal of Medicine* **38**, 1036–1041 (2021).
65. Breslin, N. *et al.* Coronavirus disease 2019 infection among asymptomatic and symptomatic pregnant women: two weeks of confirmed presentations to an affiliated pair of New York City hospitals. *American Journal of Obstetrics and Gynecology MFM* **2**, 1–7 (2020).
66. Grechukhina, O. *et al.* Coronavirus disease 2019 pregnancy outcomes in a racially and ethnically diverse population. *American Journal of Obstetrics and Gynecology MFM* **2**, 1–11 (2020).
67. Pereira, A. *et al.* Clinical course of coronavirus disease-2019 in pregnancy. *Acta Obstetricia et Gynecologica Scandinavica* **99**, 839–847 (2020).
68. Yan, J. *et al.* Coronavirus disease 2019 in pregnant women: a report based on 116 cases. *American Journal of Obstetrics and Gynecology* **223**, 111.e1-111.e14 (2020).
69. Qiancheng, X. *et al.* Coronavirus disease 2019 in pregnancy. *International Journal of Infectious Diseases* **95**, 376–383 (2020).
70. Askary, E. *et al.* Coronavirus disease 2019 (COVID-19) manifestations during pregnancy in all three trimesters: A case series. *International Journal of Reproductive BioMedicine* **19**, 191–204 (2021).
71. Sentilhes, L. *et al.* Coronavirus disease 2019 in pregnancy was associated with maternal morbidity and preterm birth. *American Journal of Obstetrics and Gynecology* **223**, 914.e1-914.e15 (2020).
72. Wu, Y. *et al.* Coronavirus disease 2019 among pregnant Chinese women: case series data on the safety of vaginal birth and breastfeeding. *BJOG: An International Journal of Obstetrics and Gynaecology* **127**, 1109–1115 (2020).
73. Conti, M. G. *et al.* Consequences of early separation of maternal-newborn dyad in neonates born to sars-cov-2 positive mothers: An observational study. *International Journal of Environmental Research and Public Health* **18**, 5899–5999 (2021).
74. Kusari Basu, J. & Chauke, L. Clinical Features and Outcomes of COVID-19 Infection among Pregnant Women in South Africa. *International Journal of Maternal and Child Health and AIDS* **10**, 174–182 (2021).
75. Bozkurt, F., Coskun, O., Bekcibasi, M., Asena, M. & Bagli, I. Comparison of Clinical and Laboratory Findings in COVID-19 Positive Pregnancies without Comborbidity. *Turkish Journal of medical Sciences* 51–468 (2021) doi:10.3906/sag-2105-116.
76. Al-Matary, A. *et al.* Clinical outcomes of maternal and neonate with COVID-19 infection – Multicenter study in Saudi Arabia. *Journal of Infection and Public Health* **14**, 702–708 (2021).
77. Amini Moghadam, S. *et al.* Clinical features of pregnant women in Iran who died due to COVID-19. *International Journal of Gynecology and Obstetrics* **152**, 215–219 (2021).
78. Gajbhiye, R. *et al.* Clinical characteristics, outcomes, & mortality in pregnant women with COVID-19 in Maharashtra, India: Results from PregCovid registry. *Indian Journal of Medical Research* **153**, 629–636 (2021).
